# Young people’s views on the acceptability and feasibility of loneliness interventions for their age group

**DOI:** 10.1186/s12888-024-05751-x

**Published:** 2024-04-23

**Authors:** Sharon Eager, Sonia Johnson, Alexandra Pitman, Manuela Uribe, Pamela Qualter, Eiluned Pearce

**Affiliations:** 1 Division of Psychiatry, University College London, Maple House, 149 Tottenham Court Road, W1T 7NF London, UK; 2 Camden and Islington NHS Foundation Trust, London, UK; 3 Manchester Institute of Education, University of Manchester, Manchester, UK

**Keywords:** Loneliness, Young adults, Acceptability, Feasibility, Adolescents, Interventions, Strategies, Interview

## Abstract

**Background:**

Loneliness is a frequently reported problem for young people aged 16 to 24 years old. A variety of interventions have been developed (but in most cases not extensively evaluated) to try and tackle loneliness in this age group. These include interpersonal, intrapersonal, and social approaches that vary in their content and mechanisms of action. The current study aimed to qualitatively examine young peoples’ views on the acceptability and feasibility of different loneliness interventions.

**Methods:**

Young people from 16 to 24 years old living in the United Kingdom who self-identified as having experienced loneliness were recruited to participate in this study. We conducted semi-structured qualitative interviews to assess their views on the acceptability and feasibility of loneliness interventions for their age group. Interviews were analysed using inductive reflexive thematic analysis.

**Results:**

Our analysis of 23 individual interview transcripts identified six themes. These related to the appropriate stage for intervention and how different types of strategies may be best suited to particular contexts; the key facilitators and barriers to engaging young people in an intervention; considerations for optimising the delivery of an intervention; divergent views on technology use in strategies to manage loneliness; the scope of an intervention and whether it takes a targeted or general approach; and the idea of combining different options within an intervention to allow tailoring to individual preferences and nature of loneliness.

**Conclusions:**

These findings demonstrate the need for continued development of individualised interventions designed to help manage loneliness in this age group. Future loneliness strategies should be co-produced with young people to ensure that they suit the varying needs of this population.

**Supplementary Information:**

The online version contains supplementary material available at 10.1186/s12888-024-05751-x.

## Background

Loneliness is an unpleasant and often distressing experience that occurs when a person feels that their social relationships are deficient in terms of quality and/or quantity [1]. It is a varied experience that has been described as manifesting in two ways: social loneliness, which results from the absence of a satisfying social network, and emotional loneliness, which is caused by a lack of intimate personal connection with others [2, 3]. Loneliness is often seen as a universal human experience, especially at key transitions and following losses [4, 5]. However, loneliness can also develop into a chronic problem, whereby individuals consistently feel that their relationships are not meaningful or satisfying [6]. This is associated with a range of adverse physical and mental health outcomes [7].

The association between loneliness and health problems applies across different age groups [8–10]. In children, adolescents, and young adults, loneliness is associated with both present and future mental health problems [11]. A consistent association has been demonstrated between loneliness and depression in the general population [12] and among young people specifically [11, 13, 14]. Recent evidence suggests that the odds of developing depression for people who are lonely may be over double that of those who are not [15]. Loneliness is also a predictor of the later development of anxiety disorders [16], and greater loneliness appears to be associated with more severe depression and anxiety symptomatology [17]. For young people with existing mental health problems, loneliness is linked with greater severity of mental health symptoms [18], in a potentially bi-directional relationship [19]. It also predicts later suicidal ideation and behaviour, particularly in young and older adult populations [20], as well as general mortality [6].

Such findings have prompted the suggestion that loneliness should be considered a public health issue [21, 22]. This may be particularly pertinent for those in adolescence and young adulthood, as the prevalence of loneliness in this age group is particularly high compared to the general population [23–25]. In 2018, young people in the UK aged 16 to 24 years old reported a higher prevalence of frequent loneliness than any other age group in the population [26]. Importantly, adolescence and young adulthood is also the developmental stage at which most mental health conditions arise [27]. Furthermore, young people were particularly vulnerable to loneliness during the recent COVID-19 pandemic compared to other population groups, potentially exacerbating this pressing issue [28–30].

Considering the above factors, and the value of early intervention, it is important to develop evidence-based interventions to tackle loneliness in young people [31, 32]. Such interventions should be developed to target loneliness as the primary issue rather than a secondary outcome, as is often the case [33]. However, until recently the majority of loneliness research investigating what may cause the problem, and/or ways to resolve it, has been conducted with older adult populations [34, 35].

Given the variation in vulnerabilities, triggers, and overall experiences of loneliness across different life stages [23] it is important that interventions should be devised with a target age group in mind [25]. This ensures that interventions are both feasible (suitable and practical to deliver) and acceptable (appropriate and satisfying for those using them) [36, 37]. These are key considerations that underline the value of co-producing research with young people into how best to help them with loneliness [22]. This may be particularly important for young people with minority ethnic identities, minority genders, and sexual minorities, as these factors appear to be associated with increased loneliness [24, 38–42].

Recent reviews of interventions addressing loneliness have aimed to target the evidence gap in this research area in relation to younger populations. Eccles and Qualter [33] conducted the first meta-analysis of evaluations of interventions that aimed to reduce loneliness targeted at those aged 3 to 25 years old, finding evidence to support effectiveness of some interventions in reducing loneliness in this age group. Although interventions designed to improve social and emotional skills had the largest effect size, the overall effect of intervention type was not significant. This suggests that different types of interventions (i.e., those that teach social and emotional skills, those that promote psychological support, and those that increase opportunities for social interaction and support) were comparably efficacious.

Another recent review in this area was conducted by Pearce and colleagues [43], who sought to evaluate loneliness interventions in terms of their designed contextual use and purported target mechanisms of action. In this review, the authors synthesised qualitative and quantitative evidence to develop a conceptual framework that identified three potential pathways to effect for loneliness interventions aimed at those aged 14 to 24 years. These comprised intrapersonal mechanisms, targeting internal psychological characteristics like low self-esteem; interpersonal mechanisms, targeting a lack of social and emotional skills; and social mechanisms, targeting a lack of emotionally fulfilling relationships.

However, both reviews identified inconsistency in the quality of the studies included in their analyses, highlighting the need for more primary evaluations of interventions before definitive conclusions can be drawn about their efficacy. In particular, it is important to identify the specific factors that young people themselves think will contribute to the success of loneliness interventions, a question best answered by using qualitative methods. This person-centred approach offers the potential to enhance the acceptability, feasibility, and efficacy of future interventions, by ensuring that they are designed to suit their target population [44].

Both reviews [33, 43] also highlight that most of the interventions identified were aimed at youth deemed to be ‘at risk’ of loneliness, such as those with autism or incarcerated youth, rather than young people who actually identify as lonely. Interestingly, another recent systematic review looking at the acceptability and effectiveness of loneliness interventions for young people aged 10 to 25 years old found that interventions designed for more general populations of young people were deemed more acceptable than those which were targeted at specific ‘at risk’ populations [45].

To add to the existing review evidence, it is important to establish what young people with lived experience of loneliness think about these interventions using qualitative methods. Direct, nuanced, and in-depth insights from young people with first-hand experiences can complement the evidence from the above quantitative reviews to allow the development of theoretical frameworks to underpin targeted interventions to reduce loneliness. Exploring the views of a range of young people in the general population who have experienced loneliness has the potential to ensure interventions can be tailored to suit a variety of individuals in this age group, not only those in specific high-risk groups.

The current study aimed to address this by tackling the question: what are young peoples’ views on the perceived acceptability and feasibility of interpersonal, intrapersonal, and social interventions to address loneliness in their age group? To answer this, we interviewed young people with experience of loneliness who were aged 16 to 24 years old, as this corresponds to the age range identified as the loneliest in the UK population [26]. Our aim was to examine their views on how acceptable and feasible different types of loneliness interventions identified in the aforementioned reviews appeared to them, and to identify any ways they thought such interventions could be optimised.

## Methods

### Study design

We used a qualitative interview study design, to assess young people’s opinions on the acceptability and feasibility of different types of interventions to help manage loneliness. We conducted individual semi-structured interviews online via a video platform (Zoom), using a topic guide.

### Sampling and recruitment

The target group for this study were young people aged between 16 and 24 years, who self-identified as having either past or current experience of loneliness and who were living in the UK at the time of the interview. We aimed to recruit up to 24 participants, to allow us to interview young people with a variety of demographic characteristics. Participants were screened using an online survey prior to the interview in which they were asked to provide their age, gender, ethnicity, sexual orientation, the type of area they lived, and whether they had experience using mental health services, collected under a randomly assigned ID code and securely stored separately to participant names and contact information. Aside from age, which was a key inclusion criterion, all demographic questions in the survey included a ‘prefer not to say’ option, so participants did not have to provide any information that they did not wish to. We employed convenience and snowball sampling methods to recruit people online, whereby individuals were invited to email the research team if they were interested in learning more about the study.

A recruitment poster was shared to Twitter, Facebook, and Instagram via institutional and individual accounts. The recruitment materials were also distributed via email lists by the UK Research and Innovation (UKRI) funded Loneliness and Social Isolation in Mental Health Research Network (LSIMHRN) and organisations in partnership with the LSIMHRN, including the Mental Elf, McPin Foundation, Tackling Loneliness Hub, and Campaign to End Loneliness.

### Study materials

We created a PowerPoint presentation to give interviewees relevant background information about the study at the start of the interview, following the consent procedure. We then presented a brief summary of each of the three types of loneliness interventions, as identified in previous reviews [33, 43], to stimulate participant reflections. These included: (i) interpersonal interventions, to improve social and emotional skills; (ii) social interventions, to improve opportunities for social interaction and support; and (iii) intrapersonal interventions, to address psychological factors. The presentation also included two examples of each of these intervention types, as featured in two recent reviews of loneliness interventions for young people [33, 43] (Additional file 1). Participants were informed that these examples were used only as illustrative representations of each of the three outlined approaches. Each intervention type and the corresponding examples were directly followed by a set of related questions, before the next intervention type was presented.

The topic guide was collaboratively developed among all members of the research team and was informed by existing research on this topic [33, 43]. Questions and prompts related to the acceptability and feasibility of each intervention type, as well as some broader concluding questions to assess participants’ general opinions on the topic (Additional file 2). The interviewees were asked not to specifically reflect on the examples given, but on the broader intervention types that they represented. To enhance validity, the study materials were reviewed by two young people who had previously participated in a lived experience group identifying potential active ingredients in loneliness interventions for young people, prior to starting recruitment. Materials were modified accordingly to ensure that they were accessible, comprehensive, and sensitive to the topic being discussed.

### Data collection

We sent participants an information sheet and consent form to review a few days prior to the interview and invited them to ask questions about these. At the start of each interview the interviewer assessed each participant’s capacity to give informed consent by checking their understanding of the study, to ensure that they had read and understood the information sheet. Consent was recorded through verbal confirmation of each consent statement.

Interviews were conducted in two stages. Fifteen interviews were conducted between the 2nd– 22nd July 2021, which was towards the end of the social distancing measures imposed by the COVID-19 pandemic. This necessitated that all interviews were conducted online. Following these interviews, we purposively recruited male, non-binary, and transgender-identifying participants, as the initial sample of 15 predominantly included female participants (73% were female). Purposive sampling resulted in eight additional interviews, which were conducted between 1st April 2022 and the 15th July 2022. All 23 interviews were carried out by one researcher (SE), using Zoom to videocall participants and record the audio. Participants received a £15 e-voucher to compensate them for their time.

### Distress protocol

The team developed a protocol for managing any participant distress arising during the interviews, whereby EP and one of two consultant psychiatrists on the team (SJ & AP) were available to be contacted during each interview to respond to any potential participant or interviewer distress. At the end of the discussion, and in a follow-up email, participants were offered contact information for mental health charities such as Samaritans, Shout, and Young Minds, in case they experienced related distress. We also offered interviewees the option of a follow-up email or telephone call to check in with them a few days after the study, if they thought they might become distressed following the interview. This option was taken by five participants.

### Data analysis

Interviews were recorded directly using Zoom. Audio recordings for the first 15 interviews were transcribed by a third-party transcription service, with the additional 8 interviews being transcribed by members of the research team. The transcripts were screened by SE and any potentially identifiable information was removed. Transcripts were securely stored under each participant’s randomly assigned ID code.

Facilitated by the analytic software NVivo, we analysed the interviews using reflexive thematic analysis, as per Braun and Clarke [46]. First, SE re-read the data for familiarisation following transcription. SE then systematically generated initial inductive codes across all interviews and the collated these into a primary thematic framework. MU independently second-coded two interviews, following the same inductive technique, and a high level of agreement was observed between both coders. Following discussion between SE and MU about codes that differed, the framework was adjusted accordingly. The thematic framework was reviewed and iteratively revised to add, modify, and combine additional themes and subthemes that were detected during analysis. The findings from the first 15 interviews were compared to those from the 8 that were conducted following additional purposive sampling. While minor tweaks were made to the coding structure following the analysis of these interviews, the primary thematic framework remained unchanged. Themes were finalised by SE and MU confirmed the suitability of the finalised framework by using it to analyse three interview transcripts. The final thematic framework was reviewed and approved by all members of the research team.

### Reflexivity and external validity

The team was comprised of researchers with a range of clinical and academic experience. The interviews were conducted by SE, who has previous experience interviewing adolescents with neurodevelopmental disorders in a research context. EP has a background in anthropology and experimental psychology research, and was present for the first three interviews to debrief with SE and reflect on how the interviews unfolded. SJ and AP are academic psychiatrists. SJ has a background in intervention development and evaluation, and AP has a research focus on self-harm and suicide epidemiology. Both have led a research programme on loneliness among people with mental health conditions for several years. MU has experience in research relating to loneliness and mental illness. PQ has a background in educational psychology research, with a focus on loneliness in children and young people. The multidisciplinary nature of the research team enabled a variety of viewpoints to shape each stage of the research process and reduce individual biases.

The interviewer (SE) and both researchers involved in analysis (SE and MU) were near to the age range that was the focus of this study. They also both had recent experience of transitions from/to a new university, which is one example of the challenging life transitions that were frequently discussed during interviews with participants. As the interviewer could directly relate to the experiences being discussed by participants, this may have influenced the prompts given and may have encouraged interviewees to relay their views more openly and in-depth. Similarly, the closeness in age and comparable experiences of the two coders may have influenced how the data was interpreted, potentially allowing them to identify important nuances in the findings.

## Results

### Participants

We interviewed 23 young people from a total of 33 volunteers. Nine individuals were excluded due to not meeting the inclusion criteria; seven were not in the desired age range, one did not live in the United Kingdom, and one did not identify as having had experience with loneliness. One individual could not participate due to technical problems. The interviews lasted between 37 and 62 min, with a mean length of 49 min (SD = 6.4).

The mean age of the interviewees was 21.3 years (SD = 2.3), with an age range of 16 to 24 years. Most of the group were heterosexual, lived in a city, and had experience using mental health services. Participants had a range of gender identities and were from a variety of ethnic backgrounds (Table 1).

**Table 1:** Participant characteristics

Demographic	Number of participants
**Mean age (standard deviation)**	21.3 (2.3)
**Gender**	
Male	8 (34.8%)
Female	11 (47.8%)
Non-binary	2 (8.7%)
Transgender (male)	2 (8.7%)
**Ethnicity**	
White/White British	8 (34.8%)
Black/Black British	6 (26.1%)
Asian/Asian British	5 (21.7%)
Multiple/mixed ethnicity	4 (17.4%)
**Sexual orientation**	
Heterosexual	15 (65.2%)
Homosexual	4 (17.4%)
Bisexual	2 (8.7%)
Pansexual	1 (4.3%)
Prefer not to say	1 (4.3%)
**Living area**	
City/Suburb	20 (87.0%)
Town/village	2 (8.7%)
Countryside	1 (4.3%)
**Current/prior use of mental health services**	
Yes	13 (56.5%)
No	7 (30.4%)
Prefer not to say	3 (13.0%)

Figures presented in the format N (%), unless otherwise stated

### Overview of themes

We identified six themes capturing participants’ views on the acceptability and feasibility of interventions to address loneliness for young people, and each theme consisted of several subthemes (Table 2).

**Table 2:** Themes and subthemes capturing participant views on loneliness interventions

Themes	Subthemes
1. Choosing the appropriate intervention for each stage of loneliness	1.1. Interpersonal strategies as early intervention 1.2. Social strategies for general loneliness 1.3. Intrapersonal strategies for more severe loneliness in the context of mental health problems
2. Engaging people in interventions	2.1. Facilitators: making intervention enjoyable and use of positive language 2.2. Barriers: stigma and lack of motivation
3. Optimising intervention setting and delivery	3.1. Benefits of a group setting 3.2. Flexible duration and continuity of support 3.3. Brief but regular session length
4. Divergent views on the role of technology	4.1. Greater accessibility and anonymity 4.2. Inferior quality of social interactions 4.3. The positives and negatives of social media
5. Clarity over the scope of an intervention	5.1. General approach for social loneliness 5.2. Targeted approach for emotional loneliness
6. Importance of using a combination approach	6.1. Importance of individual preferences 6.2. Combining varied aspects within a single intervention 6.3. Autonomy to choose preferred features

### Choosing the appropriate intervention for each stage/type of loneliness

This theme underlines the importance of ensuring that a strategy to address loneliness is appropriate for its target population, specifically by considering the stage and context during which such an intervention is introduced. Participants highlighted the unique factors of each broad intervention type that they felt made them especially suitable for particular contexts and populations.

Many of the interviewees believed that interpersonal strategies would be most appropriate as a method of early intervention at the beginning of adolescence by teaching skills relating to emotional intelligence and social communication in educational settings. They believed that such practical life skills were not currently given sufficient emphasis compared to academic subjects, and suggested that they should be considered just as important. One participant highlighted the potential long-term benefits of giving young people these tools:

It helps with - definitely for main relationships with others, and it’s a lifetime skill that they would bring into the workforce, so it’s quite essential to start building that from a young age. (P04, 21-year-old, female)

There was a prevailing opinion that such an intervention would be more appropriate for those of a younger age than those aged 16 to 24 years old. Participants worried that the content may be perceived as patronising by those over the age of 16 years, and suggested that it would be more suitable from the ages of 12 to 16. It was also generally agreed that introducing this to a younger age group would be more effective, as those entering adolescence and transitioning from primary to secondary school are likely to have fewer solidified social connections.

Social interventions designed to increase opportunities for interaction and support were well received by the interviewees. Many endorsed the idea of having a safe space to interact with other socially like-minded peers who could empathise with them and their situation, and felt that it could be an accessible way to tackle loneliness that has not become a chronic or severe issue:

I found that peer support can be more important than professional support a lot of the time. Especially if you sort of want to talk about stuff and you’re not ready to, it really does help. (P19, 24-year-old, male)

Some participants also thought that such an intervention might be intimidating for people struggling with more severe or chronic loneliness, who may find it hard to take the initiative to engage with it. Therefore, this intervention was considered most appropriate for participants who felt sufficiently comfortable to embrace unfamiliar social situations.

Participants had mixed views on intrapersonal interventions. Many strongly supported the reflective nature of interventions that targeted internal psychological characteristics and endorsed the idea of each individual working on their thinking patterns. However, others struggled to see how psychological strategies could help with loneliness specifically, as they felt that these strategies may not account for the role of situational factors in perpetuating more transient loneliness:

It’s not always that someone’s thinking pattern is the cause of loneliness, [it] may be that they moved away from their friends to go to university, or they don’t know how to make friends. (P11, 22-year-old, female)

Some believed that such strategies would be more beneficial for individuals suffering from more severe loneliness, particularly in the context of mental health problems:

Some people’s level of loneliness is higher than others and some people tend to feel lonely on just rare occasions, so it all depends on people who are deeply affected with loneliness. That set of people are those who would be ready [for this]. (P16, 20-year-old, male)

### Engaging people in interventions

The interviewees outlined a range of key factors that might encourage young people to engage in interventions to help with loneliness. Many participants believed that young people would be more inclined to get involved in strategies that seem fun and enjoyable. They suggested introducing activities or games which would encourage people to attend an intervention, and would also make it more effective:

I think having something that is actually going to make people interested in coming, an activity of some kind, and then I think you need to market it as that primarily, be like, ‘oh we are going to do this, but there is also space for you to talk about x, y, and z.' (P01, 22-year-old, non-binary)

The suggested activities included different sports, crafts, or watching a movie, as a way to break the ice and to offer natural conversation points for those in attendance.

The importance of the language used to describe an intervention was also discussed by several participants. It was agreed that language can play an important role in whether an individual decides to engage with an intervention or not, and that it was essential to avoid using clinical jargon or confusing abbreviations. They suggested using straightforward, easy to understand phrases that promote inclusivity and normalise the experience of loneliness:

I think it’s important that it’s not framed in a really clinical way, because loneliness is an emotion that everyone feels. And if it were framed as a mental health problem in its own right, then I think that alienates people from accessing strategies because I feel like that creates a threshold of, ‘well, am I lonely enough to use this service?’ (P05, 18-year-old, female)

In general, the interviewees preferred the idea of using positively framed phrases such as ‘making connections’ and ‘meeting new people’ compared to using the word ‘loneliness’, as it was agreed that focusing on the problem-solving aspect of such strategies would promote engagement.

Reasons why an individual may decide not to take part in an intervention to address loneliness were also considered. The stigma associated with admitting struggling with loneliness or being considered a ‘loner’ by other people was deemed to be a deterrent, with the consensus being that there can be a lack of understanding about seeking help for the problem:

A big thing is stigma. […] Very few people are gonna feel comfortable, you know, telling [their] peers, ‘Oh I feel lonely, I’m currently on a course addressing those things.’ It’s not something that’s culturally common. (P20, 20-year-old, transgender male)

Several interviewees discussed how they also found it challenging to recognise loneliness in themselves, often until the issue escalated into a wider mental health problem or until they were no longer feeling lonely. Many participants noted that before they experienced loneliness, they had had a lack of awareness around the potential mental health implications of the issue. Some also highlighted how they found the cycle of loneliness difficult to break due to not recognising these potential implications, and thus not having this additional motivation to initiate change:

Sometimes you get so used to the loneliness and the feelings associated with it that it feels safer and more comfortable than stepping out of your comfort zone and going to meet new people and having new experiences. (P06, 16-year-old, female)

### Optimising intervention setting and delivery

There were certain aspects of loneliness interventions that were seen as particularly important for maximising their effectiveness. A group setting was viewed as generally preferrable to approaches conducted individually. Participants felt that it was important for young people to know that others their age also struggle with loneliness:

If you’re in a group you know that you’re not alone and you’re potentially able to hear other people’s experiences and know that it’s not just you. (P08, 24-year-old, female)

Many interviewees felt that it was important for intervention participants to have the opportunity to build connections, to practise their communication skills, and to develop their social confidence in a compassionate environment. Others did note that individual sessions might be helpful for people who may particularly struggle to integrate into social groups:

Individual stuff might be more useful for those who struggle with like extra [severity of loneliness]. Or yeah, people who maybe come from a very different cultural background, perhaps. (P21, 20-year-old, male)

A recurring observation was that ensuring there was a primary focus or a shared interest among a like-minded group with similar social goals would enhance the likelihood of forming friendships:

In my opinion being lonely is not a good enough reason to meet up with someone and say, ‘oh, you’re lonely as well’. You need to have a focus and something in common. (P12, 23-year-old, male)

The length of an intervention was also a key topic of discussion among interviewees. Many participants favoured a flexible approach to the duration of an intervention, as they felt a strictly constrained number of sessions would not feasibly produce long-term change for many people. Moreover, it was noted that an intervention with a sudden end point, without some continuity of support, may not only be less effective but could also be detrimental to the individual’s progress:

I know that sometimes when it ends then people feel a bit lost, because they’ve gotten used to this community, they’ve gotten used to a support group, and then it’s kind of, removed from them. (P10, 21-year-old, female)

Some interviewees suggested that for time-limited interventions, a gradual reduction in the intensity of the intervention and additional options after its conclusion should be made available. Furthermore, they proposed that when possible, having a strategy which would be available on a drop-in, as-needed basis may be a more suitable way to tackle loneliness for this population. Many interviewees felt that this would better suit a range of people entering the strategy with different needs, to ensure that each person could make progress at their own pace. They also felt that it would be more appropriate for individuals with substantial time commitments, such as academic work or a job.

Several participants also felt that offering sessions that were brief but regular was important to achieve the best results from an intervention. Participants believed that not overloading young people with too much information at once and keeping an intervention to an hour or less to maintain the group’s attention would promote a more successful strategy. They felt that weekly sessions would ensure steady progress could be made without overwhelming those in attendance:

I think it’s good to recognise that it’s not something you can combat overnight and so making it like a weekly strategy shows that it’s not going to happen overnight. If you give yourself a goal to improve over a certain amount of time that would be really beneficial to see your progress and the change in yourself. (P15, 19-year-old, female)

This was also seen as a way to support people struggling with loneliness to different degrees, to ensure that individuals could elect to attend sessions for as long as would be helpful for them.

### Divergent views on the role of technology

Technology and its potential role in interventions for managing loneliness was a topic that produced mixed views among interviewees. Some participants endorsed instances where technological components were integrated within certain strategies, while others felt that the use of technology should be approached with caution. It was widely acknowledged that technology plays a central role in the lives of young people aged between 16 and 24 years, and thus could be a valuable tool to aid these strategies. Some participants felt that receiving an intervention remotely carried certain advantages, such as greater accessibility and a potentially less intimidating environment, factors that may increase engagement. They specifically endorsed the additional layer of anonymity offered by virtual interactions, such as online messaging:

In a sense you can say whatever you want to it kind of venting, and you won’t feel like you’re exposing yourself to other people. (P06, 16-year-old, female)

Others discussed the unique challenges posed by conducting interventions remotely, a consideration that was of particular relevance at the time due to the social distancing measures imposed by the COVID-19 pandemic. The primary concern expressed by several participants was that remote interactions, including both messaging and video calling, do not offer the same social fulfilment as in-person interactions:

I wouldn’t say necessarily speaking to someone online in any way, shape, or form is comparable to speaking to people in person. So the quality of that interaction could be massively diminished if they were to do it in online. It almost kind of defeats the purpose of going out of [one’s] way, especially if you kind of struggle, to see people, but then feel unfulfilled that you haven’t really seen them. (P20, 20-year-old, transgender male)

Some liked the idea of using an app designed to help combat loneliness, suggesting that young people would find it easy to integrate into their lives owing to their familiarity with such technology. It was further suggested that the consistent availability of an app would be convenient for people to check in with at any time that suited them. However, many participants questioned how effective an app would be to help with loneliness. Most believed that face-to-face interaction with other people was an essential component in helping with the problem, and the idea of having such an option on an app drew comparisons to social media, which was generally viewed unfavourably by participants. Many implicated social media as perpetuating loneliness in this age group, believing it to be a source of constant comparison with peers and isolation through disingenuous relationships:

I think in this age group social media’s a big influence, everyone shows the best version of themselves, so it is hard to feel like you’re the only one from what you see out there. (P15, 19-year-old, female)

Some participants did feel, however, that social media could be advantageous for promoting an intervention and reaching a wide range of young people. They felt that lonely individuals who may be hard to reach via other methods may be likely to use and respond to information posted on social media. This was also thought of as a way of normalising seeking help for loneliness and emphasising the relevance and modernity of such options.

### Clarity over the scope of an intervention

Many interviewees felt that a key consideration when developing a loneliness intervention was to clearly identify its scope and whether the aim is to reach as many people as possible or to target only those who are experiencing loneliness most frequently and intensely.

Most participants felt that for individuals who struggle with a social network with which they are dissatisfied, i.e. social loneliness, a general approach that aims to include people with a range of loneliness severity would be particularly helpful. They suggested that these individuals could benefit from working on feeling more socially connected among a diverse range of people, and proposed covering topics such as discrimination and treating everyone with respect, rather than solely on making friends. They also emphasised the potential preventative benefit of encouraging people who may not be acutely lonely to recognise loneliness in themselves and others:

There might [not] be something wrong now, per se, but when something does happen you know how to support yourself or prevent it, or support somebody else. (P03, 24-year-old, female)

Many of the participants were also keen to point out that unavoidable contextual factors may play a role in triggering and perpetuating feelings of social loneliness in young people. They discussed the many life transitions that this age group may go through, for example the transition from secondary education to further education or the workforce, and how the associated social upheaval may cause feelings of loneliness. These participants suggested that general, wide-reaching strategies to respond to such life transitions, for instance early in university, would be effective in helping people who may suddenly find themselves struggling with loneliness relating to an absent social network:

I know first-hand how difficult that transition can be in terms of like I went to a different Uni than all of my school friends. And at the time I was very quiet, very shy, that kind of [wide-reaching strategy] would have probably really helped. (P08, 24-year-old, female)

Some participants, however, felt that an approach targeted only at those with more severe loneliness was also warranted in certain situations. They suggested that individuals who felt emotionally lonely within existing social networks may generally be a harder group to reach and engage in loneliness interventions, indicating that a targeted strategy that emphasises the potential long-term consequences of severe loneliness would be more suitable for them:

I was lonely but I wasn’t fully aware of the consequences of being lonely and if I understood those consequences, I’d be more motivated to take urgent action. […] You have a deep sense of loneliness, but you are like, ‘oh, it’s passing’, or, ‘oh, I can suppress it down’, or, ‘oh’ I’m a man, I’m a guy, I don’t need to show my emotion.’ (P12, 23-year-old, male)

Furthermore, several participants emphasised that for individuals suffering from this type of prolonged loneliness, a more nuanced approach may be justified to identify specific aspects that may have caused or may be maintaining the problem:

You need to talk as a young person around why is it that you’re lonely or do you see yourself as someone who is in that cycle of loneliness, and how can you break from that cycle of loneliness? (P14, 21-year-old, male)

Several interviewees suggested that focused guidance on improving existing relationships that may be emotionally unfulfilling within these individuals’ lives, rather than on making new friendships or meeting new people, would be beneficial for those feeling emotionally lonely.

### A combination approach

An idea discussed by nearly all interviewees was that people are likely to have different preferences when it comes to interventions for loneliness. Context, communication style, and individual preference may shape responses to potential strategies. This fuelled the idea of having a multi-faceted approach to loneliness interventions, to include aspects to suit a variety of individual preferences. For instance, the idea of having both group and individual components to a strategy was discussed, to meet the needs of a wider group of people and to potentially push people out of their comfort zone:

I think that that’s meeting various people’s needs; whether they’re group focused kind of people or whether they are an individual [kind of person], it’s giving them that option but it’s also helping them learn things in different settings. (P02, 23-year-old, female)

It was suggested that this could extend to other aspects of strategies including combining in-person and online aspects, or merging different types of strategies. Some participants believed that each of the three types of loneliness interventions outlined had their individual merits and aspects that were missing. This led to the suggestion that core aspects of each could be combined to represent a more comprehensive intervention, which targets loneliness from multiple angles.

However, other participants worried that combining different strategies could make an intervention too complicated. They struggled to see how the contrasting aspects could be integrated into a coherent strategy:

I don’t think they should be combined at all, because they have different purposes, different expectations. (P09, 24-year-old, male)

Alternately, one suggestion was to arrange the different intervention types into a hierarchy of support, or stepped approach, whereby individuals could avail of additional elements of support if one strategy was not working for them:

They could be maybe in kind of like a hierarchy. […] If I make use of the first [type of] strategy and it doesn’t work for me, I can also make use of the second one, and if it doesn’t work for me I can make use of the third one. (P18, 22-year-old, non-binary)

In general, participants emphasised the importance of understanding that everyone’s experience of loneliness and personal needs were likely to be unique:

I think the one important thing with loneliness is to realise that loneliness can mean different things to different people, and loneliness for one person can be completely the opposite for the other. (P02, 23-year-old, female)

They concluded that a flexible, potentially modifiable approach that accounts for the type of loneliness and the individual needs and priorities of each person at its core would be the most successful strategy. They believed that introducing autonomy, where possible, so that individuals could prioritise their preferred aspects of an intervention would be well received by participants, likely promoting both engagement and effectiveness.

## Discussion

### Main findings

Participants expressed a wide range of opinions on the acceptability and feasibility of loneliness interventions for young people. We identified six themes that captured interviewees’ views on interpersonal, intrapersonal, and social interventions, and the specific aspects that they considered to be most critical to success. Participants felt that different intervention types were best suited to different contexts and highlighted the importance of ensuring that a strategy is appropriate for the target group. For instance, they believed that interpersonal strategies would be more suitable as early intervention for those under the age of 16 years, that social strategies are better for individuals who are mild-to-moderately lonely, whereas intrapersonal strategies would be preferable for individuals with more severe loneliness in the context of wider mental health struggles.

Participants felt that one of the big challenges with such interventions related to successfully engaging young people in them. They emphasised the importance of making strategies enjoyable by introducing fun activities and using positive language, as well as having a concerted focus on reducing the stigma often associated with loneliness. Interviewees generally felt that interventions would be most successful when including a group component and brief but regular meetings that prioritised continuity of support. They also underlined the importance of co-producing loneliness interventions with young people, to make sure they are relevant, appealing, and effective for their target group.

There was a mixed view of technology and its potential role in loneliness interventions. Participants acknowledged that it can be useful for increasing accessibility and engagement, but questioned the quality of online interactions and expressed a particular wariness about social media and the role it may play in perpetuating loneliness. They also emphasised the importance of flexibility and choice within loneliness interventions so that they can cater to a variety of preferences.

Identifying the scope of an intervention was also outlined as a key consideration, with participants suggesting that more universally applied interventions would suit those with social loneliness, while a more targeted approach may be necessary to engage individuals suffering more severely from emotional loneliness. They endorsed the idea of a more wide-reaching intervention involving people who may not be currently lonely, as a means of potentially helping to prevent future loneliness. In particular, they believed that increasing awareness about loneliness and the negative outcomes associated with it, among both actively lonely and non-lonely young people, would be beneficial to help reduce and prevent the problem.

### Findings in the context of other studies

Interviewees believed, in general, that the outlined intervention types could be helpful in combating and possibly preventing loneliness in their age group, which corroborates and is consistent with the quantitative evidence demonstrating their effectiveness [33, 45]. Building on this, participants in our study identified the specific contexts that they considered to be the most appropriate for each intervention type, and the individual factors they believed were likely to contribute to the success or failure of such strategies. This is particularly interesting given that the meta-analysis by Eccles and Qualter [33] did not find any intervention type to be significantly more effective than another, which may suggest that the efficacy of these interventions could be improved by tailoring them to young people’s needs, preferences and circumstances. Co-produced interventions are, thus, likely to be most effective, and future work should explore this possibility. The importance of lived experience input is underlined by the findings of Pearce and colleagues [43], who identified a co-designed approach to be key in the development of loneliness interventions for this population.

Another critical consideration identified by both reviews is that loneliness strategies should be flexible and personalised, a recommendation which was consistent with our findings. Interviewees stressed that personal needs and preferences should be integrated into such strategies, to develop an approach which is the most useful for each individual. Participants also discussed the specific merits of a combination approach to loneliness, whereby different elements of existing strategies could be integrated into a more comprehensive intervention. Specifically, they introduced the idea of a hierarchical or stepped approach to tackling loneliness, to enable those receiving an intervention to avail of additional supports if one intervention was not effective or suitable for them. Such proposals provide useful elaboration into how strategies can be effectively individualised while maintaining fidelity to the key components of different intervention types.

Participants highlighted the value of both specific, targeted interventions and more general, wide-reaching interventions aimed across the population of young people. This is consistent with a recent systematic review on this topic [45], which emphasised the importance of reaching a wide audience of young people to raise awareness of the issue in this population. This review also noted the role for targeted, individual level approaches alongside a wider, general approach to account for the complexity of loneliness, again consistent with the findings in our study.

Our finding that many participants had reservations about the use of technology in these interventions is interesting given the common assumption that adolescents and young adults will be readily receptive to interventions that integrate digital components [47, 48]. Interestingly, participants endorsed the use of technology and particularly social media to increase awareness about loneliness and related interventions, but were generally resistant to an intervention itself being entirely online. The mixed view of social media and its complex relationship with loneliness has been similarly highlighted in qualitative research with adolescents and young adults about their experiences of loneliness [19].

Furthermore, interviewees’ opinions on engaging young people in loneliness interventions were consistent with evidence offered by similar populations in other qualitative studies. For example, Sundqvist and Hemberg [49] asked a slightly older group, aged 17–28, for more general views on loneliness and how best to alleviate it. These participants also proposed the idea of strategies being enjoyable through the introduction of activities to foster a sense of community, and identified the importance of using positive, normalising, and problem-solving language.

### Strengths and limitations

Including input from a diverse range of participants in terms of gender, sexuality, and ethnicity is a notable strength of this study, particularly given that loneliness appears to be associated with gender, sexuality, and ethnic identity in young adults [24, 38–42]. The experience that many participants had of using mental health services may add relevance to the recommendations made, where they apply to young people experiencing loneliness alongside mental ill-health.

We included participants who had direct experience of loneliness, which importantly addresses a limitation of existing research into loneliness interventions highlighted in previous reviews [33, 43]. The inclusion of those with lived experience of loneliness, rather than participants deemed ‘at-risk’ of loneliness, provides more relevant insights the acceptability, feasibility, and potential efficacy of these interventions. The patient and public involvement during the development of study materials also likely improved the validity of these materials and ensured that they were appropriate for the target group.

It is important to note several limitations of our study. Subjectivity of interpretation during the analytic process may have introduced bias into the results. However, we mitigated this via input from a second independent coder and through the varied perspectives offered by the wider research group when iteratively developing themes. Due to the nature of loneliness, we acknowledge that young people who were severely lonely at the time of recruitment or who were uncomfortable disclosing experiences of loneliness are unlikely to have participated. This may mean that the sample did not reflect the whole range of loneliness experiences.

The majority of interviews were carried out towards the end of the social distancing measures imposed by the COVID-19 pandemic. This may have influenced participants’ views on loneliness, which was particularly heightened for young people during this time [28, 30]. It may also have influenced participants’ opinions on technology in relation to the interventions discussed, as technology use substantially increased during this time [50]. As study recruitment and interviews were conducted online due to these restrictions, it is also likely that digitally excluded people are not part of our sample. Furthermore, we acknowledge that our sample only included one participant from a rural setting, thereby underrepresenting youth in remote areas and potentially those where internet connectivity is reduced. As their views were not represented well in our study, this may reduce the resonance of our findings to young people from digitally excluded and rural settings.

### Implications

Our findings provide in-depth insights into what young people who have first-hand experience of loneliness think about the interventions available to help manage this problem. Their suggestion that different intervention types could be more effective if implemented at varying stages of loneliness has implications for future research and policy, as the utility of each intervention type may change according to these different contexts. Additional qualitative and quantitative research should explore the needs of those in late childhood and early adolescence. Future research should aim to integrate insights on loneliness interventions from digitally excluded populations.

It is important for policymakers to consider the scope of an intervention and whether a wide-reaching or targeted approach is warranted depending on the group they are aiming to reach. It may also be useful to consider a wide-reaching public health message highlighting the problem of loneliness and the potential negative outcomes associated with it, to raise awareness among both actively lonely and non-lonely young people.

The specific recommendations made by participants are important to inform the continued development, implementation, and evaluation of novel interventions that are aimed at managing loneliness in this population. This is in line with the Medical Research Council (MRC) recommended framework on the development of complex interventions [51]. Future research should build on this by specifically seeking to evaluate strategies which prioritise flexibility as well as autonomy to suit the multiple needs and preferences of their target population.

## Conclusion

Loneliness is an important and pressing issue for young people, and developing acceptable and feasible interventions for this population should be a current research priority. Co-producing future research and intervention development with insights from young people with lived experience of loneliness should be considered an essential part of this process. Specifically, those designing interventions should consider the appropriate stage and scope of an intervention, how an intervention is delivered and the role of technology, and the importance of tailoring an intervention to meet a variety of needs.

### Electronic supplementary material

Below is the link to the electronic supplementary material.

Supplementary Material 1

Supplementary Material 2

## Data Availability

The datasets used and analysed during the current study are available from the corresponding author on reasonable request.

## Abbreviations

UCL: University College London
UKRI: United Kingdom Research and Innovation
LSIMHRN: Loneliness and Social Isolation in Mental Health Research Network
SD: standard deviation
MRC: Medical Research Council
