# Supplementary material for: Young people’s views on the acceptability and feasibility of loneliness interventions for their age group
Source: BMC Psychiatry. 2024 Apr 23;24:308. doi: 10.1186/s12888-024-05751-x (PMC11040932; doi:10.1186/s12888-024-05751-x)
Supplement: Supplementary file 1 — Supplementary Material 1 [file 12888_2024_5751_MOESM1_ESM.docx]

**Additional file 1: Example interventions included to represent each intervention type**

***Interpersonal interventions:***

1. *‘Friendly Schools Project’*

Cross D, Shaw T, Epstein M, Pearce N, Barnes A, Burns S, Waters S, Lester L, Runions K. Impact of the Friendly Schools whole‐school intervention on transition to secondary school and adolescent bullying behaviour. European Journal of Education. 2018 Dec;53(4):495-513.

*Description:* A whole-school strategy to reduce frequent bullying and positively influence common mediators of bullying by encouraging students’ normative beliefs about non-acceptance of bullying, increasing feelings of support from school staff and peers if bullied, improving empathy and social competence, increasing reciprocated friendships, increasing feelings of school-connectedness, and reducing problem behaviours, absenteeism and loneliness.

1. *‘Skills for Social and Academic Success’*

Masia-Warner C, Klein RG, Dent HC, Fisher PH, Alvir J, Albano AM, Guardino M. School-based intervention for adolescents with social anxiety disorder: Results of a controlled study. Journal of abnormal child psychology. 2005 Dec;33(6):707-22.

*Description:* 14-session school-based programme focused on psychoeducation, realistic thinking, social skills training, exposure, relapse prevention, as well as unstructured social activities.

***Social interventions:***

*Digital Smartphone App ‘+Connect’:*

Lim MH, Rodebaugh TL, Eres R, Long KM, Penn DL, Gleeson JF. A pilot digital intervention targeting loneliness in youth mental health. Frontiers in psychiatry. 2019 Aug 23;10:604.

*Description:* 6-week smartphone intervention using a strengths-based positive psychology framework. Delivers videos and posts daily in an attempt to convey evidence-based concepts known to strengthen relationships and increase social connections.

*University peer social support group:*

Mattanah JF, Ayers JF, Brand BL, Brooks LJ, Quimby JL, McNary SW. A social support intervention to ease the college transition: Exploring main effects and moderators. Journal of college student development. 2010;51(1):93-108.

*Description:* 9-week social support group during first year of college. 6-10 students in each with semi-structured training guide used by the group facilitators. Discussions around creating new social ties, balancing academic and social life, peer pressure, values, college life, residential issues, expectations vs realities of college, and maintaining existing relationships. Sessions lasted for approximately 90 minutes.

***Intrapersonal interventions:***

*Group mindfulness training*

Zhang N, Fan FM, Huang SY, Rodriguez MA. Mindfulness training for loneliness among Chinese college students: A pilot randomized controlled trial. International Journal of Psychology. 2018 Oct;53(5):373-8.

*Description:* 8 weekly 2-hour on-campus group sessions, focused on loneliness psychoeducation, learning theories, practicing mindfulness exercises, and discussing home practice.

*Group cognitive behavioural therapy:*

Stice E, Rohde P, Seeley JR, Gau JM. Brief cognitive-behavioral depression prevention program for high-risk adolescents outperforms two alternative interventions: a randomized efficacy trial. Journal of consulting and clinical psychology. 2008 Aug;76(4):595.

*Description:* 6 weekly 1-hour sessions focused on building group rapport, increasing involvement in pleasant activities, and replacing negative cognitions with positive cognitions. In-session exercises required youth to apply the skills taught in the intervention. Homework was used to reinforce the skills taught in the sessions and help participants learn how to apply these skills to their daily life.
