# Supplementary material for: Young people’s views on the acceptability and feasibility of loneliness interventions for their age group
Source: BMC Psychiatry. 2024 Apr 23;24:308. doi: 10.1186/s12888-024-05751-x (PMC11040932; doi:10.1186/s12888-024-05751-x)
Supplement: Supplementary file 2 — Supplementary Material 2 [file 12888_2024_5751_MOESM2_ESM.docx]

**Additional file 2: Interview topic guide**

*Present and discuss slides on ‘strategies to improve opportunities for social interaction and support’ (12 mins)*

- What are your initial thoughts on this type of strategy?
  - Can you identify any specific pros and cons of this type of approach?
- Are there any aspects of this approach that you particularly liked or disliked the sound of?
  - Reasons?
- Do you think this type of strategy would be successful for young people of your age?
  - Reasons?
  - Better in an online format or in person?
  - Better delivered as part of a group or as an individual?
  - Where would it be best to deliver the strategy and recruit from?
  - What length of strategy do you think would work best?
- Is there anything that you would change about this approach?
  - Is there anything important that it does not cover?

*Present and discuss slides on ‘strategies to improve psychological support’ (12 mins)*

- What are your initial thoughts on this type of strategy?
  - Can you identify any specific pros and cons of this type of approach?
- Are there any aspects of this approach that you particularly liked or disliked the sound of?
  - Reasons?
- Do you think this type of strategy would be successful for young people of your age?
  - Reasons?
  - Better in an online format or in person?
  - Better delivered as part of a group or as an individual?
  - Where would it be best to deliver the strategy and recruit from?
  - What length of strategy do you think would work best?
- Is there anything that you would change about this approach?
  - Is there anything important that it does not cover?

*Present and discuss slides on ‘strategies to improve social and emotional skills’ (12 mins)*

- What are your initial thoughts on this type of strategy?
  - Can you identify any specific pros and cons of this type of approach?
- Are there any aspects of this approach that you particularly liked or disliked the sound of?
  - Reasons?
- Do you think this type of strategy would be successful for young people of your age?
  - Reasons?
  - Better in an online format or in person?
  - Better delivered as part of a group or as an individual?
  - Where would it be best to deliver the strategy and recruit from?
  - What length of strategy do you think would work best?
- Is there anything that you would change about this approach?
  - Is there anything important that it does not cover?

*General concluding questions (12 mins)*

- What is the best way to get young people involved in these types of strategies?
  - In schools/universities?
  - Social media?
- What kind of language do you think is best to use in strategies like these?
  - Loneliness? Making connections? Creating belonging?
- Do you think any one type of approach sounds particularly promising?
  - Reasons?
  - Is there any that you think is particularly unlikely to work?
- Overall, is there anything that that you think all of these strategies miss out on?
  - Is there anything that has not been covered that you think is important to consider?
- Do you think different parts of these strategies could be combined, or should they be kept as separate approaches?
  - What aspects do you think are the most important to include?
